# Preparation of Self-Assembled Human Serum Albumin Nanoparticles Decorated with Trastuzumab as a Paclitaxel Delivery System

**DOI:** 10.3390/mi17010055

**Published:** 2025-12-30

**Authors:** Alexa H. Gonzalez-Posada, Yuliana Monsalve, Betty Lucy López, Ligia Sierra

**Affiliations:** Materials Science Group, Institute of Chemistry, University of Antioquia, Calle 70 #52-21, Medellín AA 1226, Colombia

**Keywords:** albumin nanoparticles, paclitaxel delivery, self-assembled nanoparticles, trastuzumab, nanocarrier design

## Abstract

This study reports the development of paclitaxel (PTX)-loaded human serum albumin (HSA) nanoparticles (NPs), surface-decorated with trastuzumab (TMAB), with potential applicability in HER2-oriented delivery. The NPs were obtained via thermally driven self-assembly followed by non-covalent antibody adsorption and they were characterized using Fourier transform infrared spectroscopy (FTIR), dynamic light scattering (DLS), and ζ-potential analysis. The drug association efficiency (*%DAE*), defined exclusively for PTX, was high for both HSA-PTX and HSA-PTX-TMAB NPs (96.4% and 98.2% *w*/*w*, respectively), with loading capacities (*%LC*) of 8.9% and 7.4%, respectively. TMAB decoration led to a modest increase in mean diameter and a reduction in surface charge, consistent with successful surface modification. Both formulations exhibited rapid early-phase PTX release followed by an apparent stabilization phase, with distinct kinetic behavior between HSA–PTX and HSA–PTX–TMAB NPs. Cytotoxicity in A549 cells after 18 h of exposure showed modest, non-differential effects consistent with controlled release and short-term assessment of non-specific toxicity. Overall, this thermally assembled albumin-based system provides a promising foundation for further evaluation of HER2-oriented PTX delivery.

## 1. Introduction

Paclitaxel (PTX) is a microtubule stabilizing agent that induces cell cycle arrest at the G2/M phase, ultimately leading to cell death. It is widely regarded as one of the most often used anticancer drugs, demonstrating efficacy against various types of cancer, such as ovarian, breast, and lung cancer [1]. However, due to its poor aqueous solubility, the commercially available liquid formulation (Taxol^®^) uses a carrier consisting of a 1:1 combination of Cremophor^®^ EL (CrEL) and dehydrated ethanol [2].

CrEL is a non-ionic solubilizer and emulsifier synthesized through the reaction of ethylene oxide with castor oil [3,4]. It appears as a pale yellow, oily liquid primarily composed of polyethylene glycol (PEG) conjugates, including fatty acid esters of PEG, hydrophilic PEGs, and ethoxylated glycerol derivatives [5]. Although effective in enhancing PTX solubility, CrEL is associated with several adverse effects including hypersensitivity reactions, peripheral neuropathy, and neurotoxicity [1,6].

To address these limitations, the recent literature describes various PTX delivery systems, such as liposomes, albumin-bound PTX nanoparticles (NPs), solid lipid nanoparticles [7,8], micelles [9,10], and dendrimers [11,12]. In particular, recent studies have focused on amphiphilic polypeptide-based nanocarriers, which exploit the self-assembly of peptide or protein-derived segments to encapsulate hydrophobic drugs [13,14,15]. These systems include synthetic polypeptides bearing hydrophobic amino acid blocks, peptide–polymer hybrids, and protein-mimetic constructs, which form micellar or nanoparticulate architectures capable of PTX loading and controlled release; these systems have demonstrated promising drug loading capacity, stability, and antitumor activity [16].

Despite their promising drug loading capacity and antitumor performance, many amphiphilic polypeptide-based systems rely on complex multistep syntheses, sequence-specific chemical modifications, which may complicate large-scale production [17]. In addition, peptide-based drug delivery systems often rely on parenteral administration [18] and require chemical or formulation-based stabilization to preserve bioactivity, which limits their clinical practicality. These constraints are largely associated with poor membrane permeability and limited in vivo stability of peptides, restricting their broader application to drug delivery [19,20].

In this context, human serum albumin (HSA), a naturally occurring polypeptide, has emerged as a promising biomaterial for drug delivery due to its intrinsic physicochemical and biological properties [21]. HSA is the most abundant and stable globular protein in human plasma, representing 52–62% of total plasma proteins, and is composed of a 66.5 kDa non-glycosylated single polypeptide chain with 585 amino acid residues [22]. It is predominantly helical and is organized in three homologous domains (I–III) [22,23], each subdivided into two subdomains, A and B. These structural features confer different binding sites for fatty acids, hormones, metal ions, antibiotics, and drugs [24]. This structural versatility, combined with high water solubility, biodegradability, biocompatibility, and low immunogenicity [21], has established HSA as an excellent carrier platform for pharmaceutical applications [25].

Accordingly, HSA has been extensively investigated as a nanotransport platform. Albumin-based NPs exhibit natural tumor accumulation due to the enhanced permeability and retention (EPR) effect, which enables preferential deposition in malignant and inflamed tissues [26,27]. Notably, this passive targeting mechanism offers a distinct advantage over peptide-based systems, the limited membrane permeability of which can restrict tissue penetration and reduce effective accumulation at the target site.

One notable example of clinical translation is the nanoparticle albumin-bound (Nab™) technology utilized in Abraxane^®^, a commercial paclitaxel formulation. The Nab™ process involves mixing organic solvents such as chloroform or dichloromethane with aqueous human serum albumin (HSA) to form a coarse emulsion, which subsequently undergoes high-pressure homogenization to yield the final NPs [28,29]. While effective, this method relies on hazardous organic solvents and intensive mechanical processing, which complicates its scalability and introduces additional safety considerations.

In contrast to Nab™, the method used in the present study relies exclusively on controlled thermal denaturation of HSA to induce protein self-assembly. This process is governed by temperature-dependent conformational transitions. Domain II unfolds reversibly below 70 °C [30] while domain I undergoes irreversible changes at higher temperatures [31], exposing non-polar residues that promote intermolecular association and NP formation [32]. The resulting assemblies are stabilized by hydrophobic interactions, hydrogen bonding, electrostatic forces, and Van der Waals interactions [33,34], which collectively support the formation of a low-energy stable matrix [35].

The structural rearrangements, typically reflected by the loss of α-helical content and increased random coils and intermolecular β-sheets, can be strategically exploited for NP engineering [36].

Ethanol is introduced to this formulation not as a desolvating agent, as in traditional albumin nanoparticle preparation, but specifically as a solvent for PTX. Its polar protic nature reduces local protein hydration and transiently modulates albumin solubility, subtly favoring the conformational environment required for PTX incorporation during self-assembly [37,38].

To improve delivery specificity, HSA NPs may be functionalized with targeting ligands [39], including monoclonal antibodies such trastuzumab (TMAB) which binds the extracellular domain of Human Epidermal Growth Factor Receptor 2 (HER2) [40,41]. TMAB not only inhibits HER2-mediated signaling, but also promotes receptor internalization and antibody-dependent cytotoxicity (ADCC) [42,43]. In this way, TMAB can be attached to the NPs’ surface via covalent or non-covalent approaches, with latter offering a simpler and milder strategy based on electrostatic and weak in-termolecular interactions [44,45].

Although albumin-based nanocarriers have advanced considerably, two critical aspects remain underexplored: (i) how the influence of the conformational state of HSA during thermal unfolding determines HSA-PTX NP formation, the *%DAE*, and the colloidal stability; and (ii) the impact of non-covalent adsorption of large biomolecules such as TMAB on the structural microenvironment of HSA and consequently its drug release behavior. Most current HSA–PTX systems utilize desolvation and chemical crosslinking [46,47], while TMAB-functionalized albumin NPs often require covalent conjugation steps that may disrupt protein architecture [48,49]. In contrast, this study establishes a thermally driven self-assembly approach in phosphate-buffer saline (PBS) and quantitatively analyzes secondary structure transitions of HSA using FTIR deconvolution, correlating these transitions with NP formation and PTX encapsulation.

Additionally, TMAB adsorption is shown to induce detectable structural perturbations in the HSA matrix and alter the PTX release mechanism.

Through the integration of conformational analysis, physicochemical characterization, and mechanistic release modeling, this study provides a formulation-driven framework for the rational design and optimization of albumin-based nanocarriers beyond traditional crosslinked or covalently modified systems.

## 2. Materials and Methods

### 2.1. Materials

The human serum albumin (lyophilized powder ≥ 97% *w*/*w* agarose gel electrophoresis, product code A9511Sigma-Aldrich, MilliporeSigma 3050 Spruce St, St. Louis, MO 63103, USA), sodium hydroxide (ACS reagent ≥ 97.0% *w*/*w*, pellets, product code 221465), fuming hydrochloric acid (ACS reagent, 37% *w*/*v* product code 258148), anhydrous ethanol (200 proof, anhydrous ≥ 99.5% *v*/*v* product code 459836), and sodium chloride (ACS reagent, ≥99.0% *w*/*w*, product code S9888) were all bought from Merck (Merck Millipere, 6000 N Teutonia Ave, Milwaukee, WI 53209, USA). The deionized water (milliQ), monobasic sodium phosphate (ReagentPlus^®^ ≥ 99.0% *w*/*w*, product code S0751), and sodium phosphate dibasic (ACS reagent ≥ 99.0% *w*/*w*, product code S9763) were purchased from Sigma Aldrich. The paclitaxel (99.92% *w*/*w* product code T0968) was purchased from TargetMol^®^ and the trastuzumab (Herceptin^®^) was purchased from Roche (Grenzacherstrasse 124, 4070 Basel, Switzerland).

### 2.2. Methods

#### 2.2.1. Preparation of HSA NPs (HSA-PTX, HSA-TMAB, and HSA-PTX-TMAB)

HSA NPs were prepared via a self-assembly method that relies on controlled thermal denaturation to promote structural changes that enable the NPs’ formation and drug encapsulation. In all formulations, 6.0 mg of HSA was dissolved in 1.0 mL of 0.16 M PBS, of pH 7.4, chosen to simulate physiological conditions and ensure PTX stability at this pH [50]. PBS was specifically selected due to its stabilizing interaction with HSA during thermal stress; phosphate ions can interact electrostatically and via hydrogen bonding with positively charged resides such as lysine and arginine, thus minimizing aggregations and preserving protein functionality under heat treatment [51].

The HSA solution was initially subjected to incubation at 25 °C for 30 min to allow for full hydration and buffer–protein interaction, followed by a second incubation period at 70 °C for 30 min to induce thermal denaturation. This temperature condition is known to cause irreversible unfolding of domain I and reversible changes in domain II, leading to the exposure of hydrophobic amino acid residues that were previously buried in the HSA core [31]. The increased hydrophobicity of the unfolded HSA facilitates the non-covalent incorporation of PTX via hydrophobic and Van der Waals interactions, with minor contributions from hydrogen bonding [52,53].

In parallel, a PTX stock solution (20 mg/mL) was prepared in ethanol by shaking for 5 min. For the formulation of the HSA-PTX NPs, 40 μL of the PTX solution was added drop by drop to the denatured HSA solution, followed by 1260 μL of deionized water at a rate of 1 mL/min under constant magnetic stirring at 500 rpm for 4 h.

To obtain the HSA-PTX-TMAB NPs, 40 μL of the PTX solution and 150 μL of a 1% TMAB solution were sequentially added to the denatured HSA, followed by 1110 µL of deionized water under the same stirring and addition conditions.

For the preparation of the HSA-TMAB NPs (control formulation without PTX), 40 µL of ethanol (without PTX) was added to the denatured HSA, followed by 150 µL of a 1% TMAB solution, and finally 1110 µL of deionized water, all under the same magnetic stirring and rate conditions.

All formulations were subsequently subjected to characterization and performance evaluation.

#### 2.2.2. Conformational Analysis of HSA by FTIR

Fourier transform infrared spectroscopy (FTIR) was used to explore the HSA conformational changes induced by thermal denaturation under the same conditions employed for NP preparation. This analysis aimed to confirm the structural rearrangements associated with exposure of the hydrophobic domains, which are critical for the self-assembly process and the drug association efficiency.

Lyophilized samples (FreeZone Freeze Dryers, LABCONCO, Labconco/FreeZone, Labconco Corporation, Kansas City, MO, 64132-2696 USA) of native and heat-treated HSA were analyzed using a Perkin Elmer Spectrum Two infrared spectrometer equipped with an attenuated total reflectance (ATR) crystal. Analyses were conducted at room temperature using 32 scans per sample, with a resolution of 4 cm^−1^ and a wavenumber range between 4000 and 600 cm^−1^.

The spectra were processed using the second derivative method to enhance band resolution, followed by deconvolution in the amide I band region (1600–1700 cm^−1^), which is sensitive to protein secondary structure. A Gaussian nonlinear fitting procedure was applied using Origin Pro 2016 software (OriginLab Corporation: One Roundhouse Plaza, Suite 303 Northampton, MA 01060, USA), in accordance with established methods [54,55].

#### 2.2.3. Physicochemical Characterization of NPs

For a drug delivery system, there are desired pharmaceutical properties, such as stability, drug release behavior, targeting, and pharmacokinetic and pharmacodynamics characteristics [56,57]. These properties are directly influenced by particle nanostructures, including hydrodynamic size, ζ-potential, storage stability, surface morphology, polydispersity index (PDI), and drug loading.

- Mean diameter and Polydispersity Index

The mean diameter and size distribution of the HSA-PTX, HSA-TMAB, and HSA-PTX-TMAB NPs were measured by dynamic light scattering (DLS) using a HORIBA LB 550 system (Kyoto, Japan). Measurements were carried out at 25 °C using aqueous dispersions of each formulation. All measurements were performed in triplicate. The polydispersity index was also recorded to evaluate the uniformity of the particle population.

- ζ-Potential

The surface charge of each NP formulation was assessed through ζ-potential analysis using a Malvern Zetasizer Nano Series instrument. Samples were diluted 1:10 with deionized water and analyzed at room temperature in triplicate. This parameter provides insight into the colloidal stability of the NP systems.

- Storage stability

The colloidal stability of the HSA-PTX and HSA-PTX-TMAB NPs was determined over time by monitoring changes in the mean NP diameter, PDI, and ζ-potential after storage at 4 °C for one month. Measurements were performed at 25 °C, at defined intervals to detect potential destabilization events like aggregation, size, or charge reduction.

- Morphological Analysis by SEM

The morphology of the HSA-PTX and HSA-PTX-TMAB NPs was analyzed by scanning electron microscopy (SEM) using a JEOL JSM-6490LV microscope (3-1-2 Musashino, Akishima-shi, Tokyo 196-8558, Japan). Lyophilized NPs (FreeZone Freeze Dryers, LABCONCO) were deposited onto graphite tape and coated with a gold layer using a Denton Vacuum Desk IV sputter coater to enhance image resolution and prevent charging. The SEM images provided visual confirmation of shape and size to complement the DLS data.

#### 2.2.4. Drug Loading and Release Studies

- Paclitaxel drug association efficiency and Loading Capacity

The drug association efficiency (*%DAE*) of the HSA-PTX and HSA-PTX-TMAB NPs was determined using ultrafiltration followed by high performance liquid chromatography (HPLC) analysis. For each formulation, 2.0 mL of the NP dispersion was transferred to an Amicon^®^ Ultra-50 centrifugal unit (MWCO 50 kDa) and centrifuged at 7780 rpm until 500 µL was collected. Then, the NPs were washed by adding 500 µL of ultrapure water and they were centrifuged again under the same conditions. The two filtrates were pooled to obtain a final volume of 1000 µL.

An aliquot of 100 µL from the collected supernatant was mixed with 100 µL of PBS (pH 7.4) and 900 µL of ACN, vortexed, filtered through 0.22 µm PTFE membranes, and analyzed by HPLC using a Waters™ (Waters Corporation, 34 Maple Street, Milford, MA 01757, USA) system equipped with a Waters™ 2489 UV/Vis detector. Chromatographic separation was carried out on a Phenomenex Luna C18 column (with a 250 mm × 4.6 mm × 5.0 µm particle size), maintained at 25 °C. The mobile consisted of an isocratic solution of ACN and water (70:30 *v*/*v*), delivered at a flow rate of 0.8 mL/min. The detection wavelength was set at 228 nm and each injection volume was 20 µL, with a total run of 10 min [58].

The quantification was based on a calibration curve prepared by diluting PTX in an ACN/PBS (90:10 *v*/*v*) mixture, covering a concentration range from 3.9 µg/mL to 250 µg/mL (seven data points). The calibration curve showed excellent linearity with a correlation coefficient (R) of 0.999998 and a coefficient of determination (R^2^) of 99.9997%. The *%DAE* and *%LC* were calculated according to Equations (1) and (2) [59]:

(1) $$\%DAE=\frac{Weight\;of\;PTX\;used\;in\;formulation-weight\;of\;PTX\;in\;supernatant}{Weight\;of\;PTX\;used\;in\;formulation}\;\times 100$$

(2) $$\%LC=\frac{weight\;of\;PTX\;in\;NPs}{Weight\;of\;recovered\;NPs}\times 100$$

All experiments were performed in triplicate.

- In vitro PTX Release

The in vitro release profile of PTX from the HSA-PTX and HSA-PTX-TMAB NPs was evaluated using a dialysis-based method. A volume of 1 milliliter of each nanoparticle formulation was transferred into a Float A Lyzer^®^ dialysis device (MWCO 20 kDa, Merck Millipere, 6000 N Teutonia Ave, Milwaukee, WI 53209, USA) and immersed in 100 mL of PBS at pH 7.4 and 37 °C under continuous stirring at 100 rpm. At predefined time intervals, 100 μL aliquots were withdrawn from the dialysis tube and immediately replaced with an equal volume of fresh PBS to maintain a constant volume and concentration gradient. Owing to the limited aqueous solubility of PTX, these experimental conditions may lead to a partial loss of sink conditions during the release process [60]. Nevertheless, this methodology was selected in accordance with previously reported in vitro release studies of albumin and PTX delivery systems, enabling direct comparison with data in the literature [61,62].

Independently of these considerations, PTX is known to be stable under these experimental conditions [63], supporting the reliability of the detected release profiles. The cumulative percentage of drug release at each time point was calculated using Equation (3):

(3) $$release\%=\left(\frac{weight\;of\;PTX\;in\;HSA-PTX\;NPs-weight\;of\;PTX\;in\;each\;aliquot}{weight\;of\;PTX\;in\;HSA-PTX\;NPs}\right)\times 100$$

The release data were analyzed using various kinetic models to understand the mechanism of drug release, including the zero-order, first-order, Higuchi, Hixson–Corwell and Korsmeyer–Peppas models [64,65].

#### 2.2.5. In Vitro Cytotoxicity Assay

An analysis of the cytotoxic effects of the HSA-PTX and HSA-PTX-TMAB NPs was conducted using the human alveolar basal epithelial cell line A549, a widely accepted in vitro model for non-small cell lung cancer (NSCLC) with overexpression of the HER2 receptor [66]. The MTT assay (3-(4,5-dimethylthiazol-2-yl)-2,5-diphenyltetrazolium bromide) was employed to assess cell viability, proliferation, and metabolic activity following NP exposure [67].

This colorimetric assay is based on the reduction of MTT by mitochondrial dehydrogenase enzymes in viable cells, producing purple formazan crystals [68]. After incubation, 200 µL of 30% dimethyl sulfoxide (DMSO) was added to each well to dissolve the formazan and the resulting colored solution was quantified by measuring absorbance at 500–600 nm using a Multiskan Go 1.01.12 Plate Reader (Thermo Scientific™, Waltham, MA, USA). The intensity of the color directly correlates to the number of metabolically active and viable cells [67].

The A549 cells were seeded in 96 well plates at a density of 20,000 cells per well and incubated for 18 h to allow for cell adhesion and recovery [69]. A 10 nM stock solution of PTX in ethanol was prepared as reference. The experimental groups included free PTX, HSA-PTX NPs, HSA-PTX-TMAB NPs, and blank formulations (without PTX), all adjusted to deliver final PTX concentrations of 10 nM and 20 Nm in the culture medium. These concentrations were selected based on the therapeutic range of PTX used in adjuvant chemotherapy for lung adenocarcinoma [70].

After dilution in the culture medium, the residual ethanol concentration was extremely low. For the 10 nM PTX condition, the final ethanol concentration was approximately 4 µg/mL (~0.0005% *v*/*v*) and for the 20 nM PTX condition, it was approximately 8 µg/mL (~0.001% *v*/*v*). These levels are several orders of magnitude below those reported to affect cell proliferation, as ethanol concentrations between 2.5% and 0.15% have been shown to be well tolerated by cultured cells without impairing viability or metabolic activity [71]. Therefore, the residual ethanol in the formulations is not expected to affect nanoparticle stability or interfere with cytotoxic outcomes.

#### 2.2.6. Statistical Analysis

All experimental data were expressed as mean ± standard deviation (SD). Unless otherwise specified, the analyses were performed using a minimum of three independent replicates; the cytotoxicity assay was conducted with technical triplicates. The statistical analysis was performed using Statgraphics Centurion XVI (Statgraphics Technologies, Inc. The Plains, VA 20198, USA). Data normality was evaluated with the Kolmogorov–Smirnov test. For normally distributed datasets, differences between groups were assessed using one-way analysis of variance (ANOVA), followed by post hoc comparisons when appropriate. For non-normal datasets, the Kruskal–Wallis test was applied. A 95% confidence level was used and differences were considered statistically significant at *p* < 0.05.

## 3. Results

**Figure 1:**
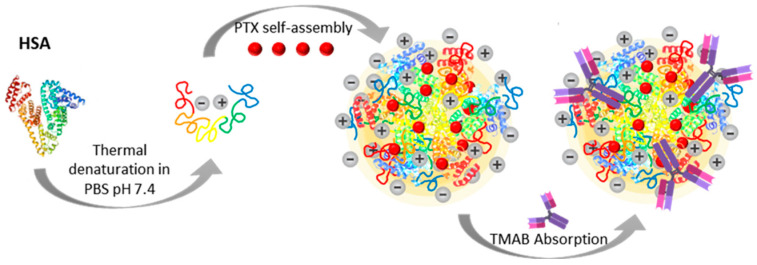
Schematic representation of the formation and structural organization of the albumin nanoparticle (NP) systems. Human serum albumin (HSA) undergoes structural rearrangement and self-assembles with paclitaxel (PTX), which is incorporated into the hydrophobic domains of the protein matrix, leading to the formation of HSA-PTX NPs. In the second system, trastuzumab (TMAB) is subsequently adsorbed onto the surface of the preformed HSA-PTX NPs through non-covalent interactions, resulting in HSA-PTX-TMAB NPs. The scheme highlights the key preparation steps and the structural differences between the non-decorated and antibody-decorated NPs. The +/− symbols represent the charges of the amino acids at pH 7.4.

The HSA-based NPs were prepared using a thermal self-assembly process that induces controlled conformational changes in HSA, facilitating nanoparticle formation and PTX association. A subsequent mild adsorption step was employed to generate the trastuzumab-decorated system (Figure 1). This section first examines the structural transitions of HSA following heat treatment, then presents the physicochemical properties of the NPs, followed by the analyses of drug association and release behavior and concludes with the functional evaluation of the formulations.

### 3.1. Structural Characterization of HSA by FTIR

The FTIR method was used to identify conformational changes in HSA resulting from thermal denaturation, correlating with variations in secondary structure. This analysis was performed through the second derivative and deconvolution of the amide I band (1600–1700 cm^−1^) and correlated with the reported values in Table 1 [72,73].

**Figure 2:**
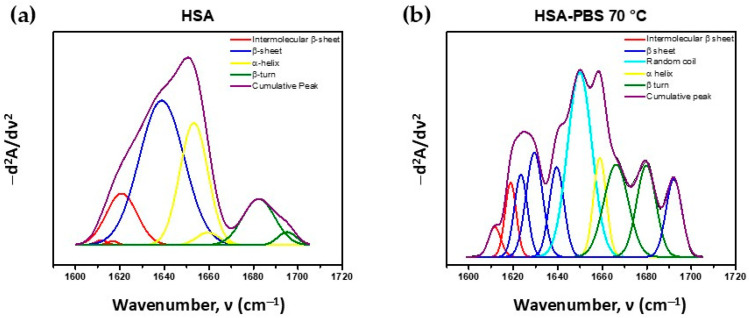
The second derivative deconvolution of amide band I; (**a**) the native human serum albumin (HSA) spectra and (**b**) the human serum albumin (HSA) spectra after denaturation in PBS pH 7.4 at 70 °C (HSA-PBS 70 °C).

Figure 2 shows spectra for (a) native HSA and (b) HSA thermally denatured in PBS at 70 °C. The native protein displayed no signal between 1640 and 1649 cm^−1^, suggesting minimal random coil content [72], and a weak band near 1610–1619 cm^−1^, indicative of limited intermolecular β-sheet formation. In contrast, thermally denatured HSA exhibited increased signal intensity in both regions, reflecting changes in the protein secondary structure.

Table 2 summarizes the relative proportions of the secondary structures obtained from deconvolution of the amide I band (1600–1700 cm^−1^).

### 3.2. Physicochemical Characterization

The physicochemical properties of the prepared NPs are summarized in Table 3. The HSA-PTX and HSA-PTX-TMAB NPs showed mean diameters of 287.1 ± 12.9 nm and 306.9 ± 42.2 nm, respectively, both with PDI values below 0.3, confirming the formation of narrowly dispersed NP populations [74].

The HSA-TMAB NPs presented the largest mean diameter (379.1 ± 3.3 nm) and the highest PDI (0.30 ± 0.23), compared to the other formulations. The ζ-potential values were −36.5 ± 1.4 mV for HSA-PTX, −21.1 ± 5.0 mV for HSA-TMAB, and −24.0 ± 1.4 mV for HSA-PTX-TMAB.

**Figure 3:**
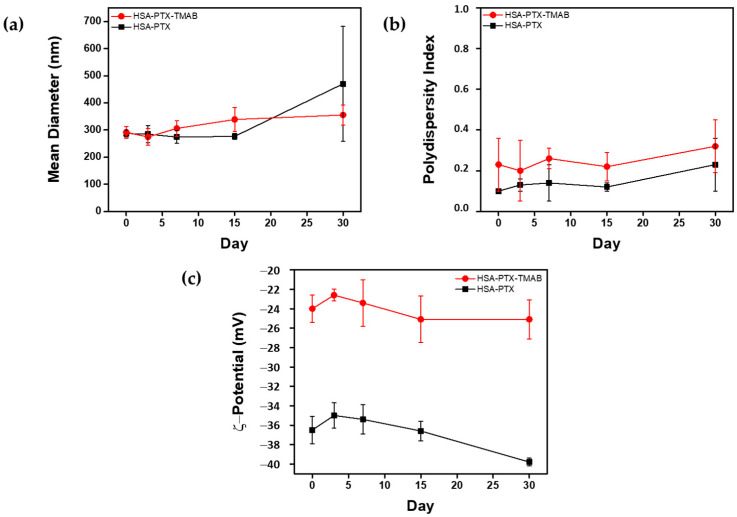
The storage stability of HSA-PTX and HSA-PTX-TMAB nanoparticles (NPs) in phosphate-buffered saline (PBS, pH 7.4) over 30 days at 4 °C. The (**a**) mean diameter, (**b**) polydispersity index (PDI), and (**c**) ζ-potential were measured at predetermined time points. The data represents the standard deviation (SD) and the error bars represent the SD among three independent replicates (*n* = 3). The normality was assessed using the Kolmogorov–Smirnov test. For normally distributed datasets, one-way ANOVA was applied. No statistically significant differences were observed over time in the mean diameter or the PDI for either formulation. For the ζ-potential, the HSA-PTX NPs exhibited significantly more negative values than the HSA-PTX-TMAB NPs at several time points (*p* < 0.05).

Figure 3 shows the stability profiles over 30 days at 4 °C. Both the HSA-PTX and HSA-PTX-TMAB formulations remained stable for 30 days, with no evidence of phase separation or aggregation. The PDI values remained constant throughout the study. ζ-potential measurements showed a slight decrease over time, particularly for HSA-PTX-TMAB, indicating minor surface rearrangements or partial desorption of TMAB.

**Figure 4:**
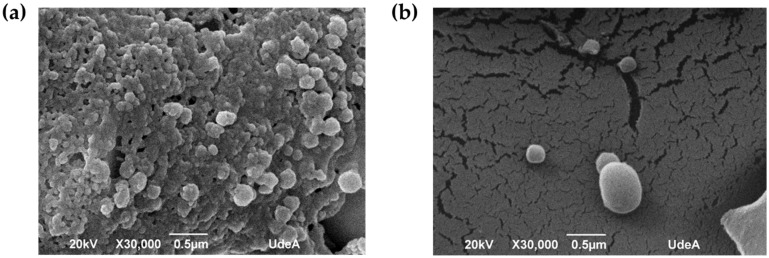
(**a**) Scanning electron microscopy (SEM) image of lyophilized human serum albumin–paclitaxel nanoparticles (HSA-PTX NPs) prepared by thermal denaturation of human serum albumin (HSA) and self-assembly with paclitaxel (PTX) (scale = 0.5 µm). (**b**) SEM image of lyophilized human serum albumin–paclitaxel–trastuzumab nanoparticles (HSA-PTX-TMAB NPs) obtained by thermal denaturation of HSA, self-assembly with PTX, and surface decoration with trastuzumab (TMAB) via the adsorption method (scale = 0.5 µm).

SEM micrographs of the HSA-PTX and HSA-PTX-TMAB NPs are shown in Figure 4. Both formulations exhibited spherical morphology with smooth surfaces and no evidence of structural collapse after lyophilization.

### 3.3. Drug Loading and Release Studies

The drug association efficiency (*%DAE*) of PTX was calculated using Equation (1) and, for the HSA-PTX and HSA-PTX-TMAB formulations, was 96.4 ± 2.1% and 98.2 ± 3.5%, respectively, with no statistically significant differences (*p* > 0.05) (Table 4). The loading capacity (*%LC*) of PTX, using Equation (2), was 8.9 ± 2.9% for HSA-PTX and 7.4 ± 1.5% for HSA-PTX-TMAB.

**Figure 5:**
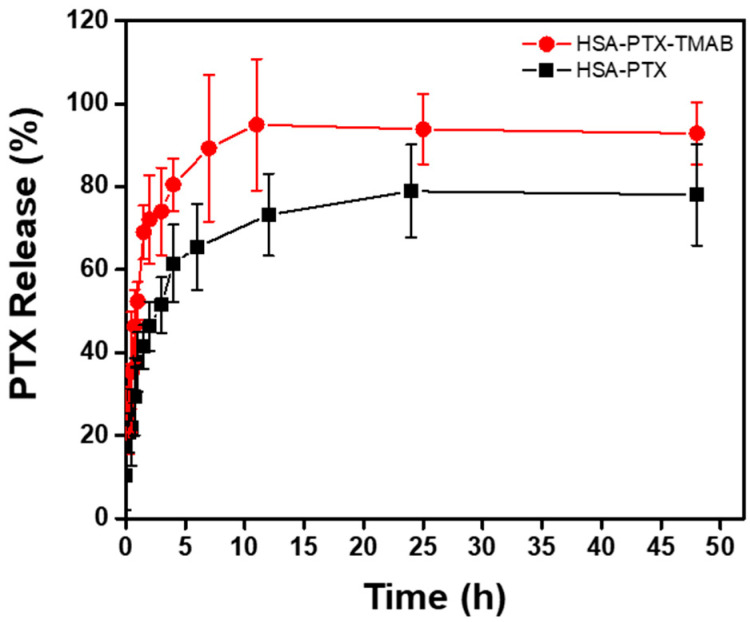
Release profile of paclitaxel (PTX) from human serum albumin nanoparticles (HSA-PTX NPs) and human serum albumin–paclitaxel–trastuzumab nanoparticles (HSA-PTX-TMAB NPs) at 37 °C and physiological pH 7.4. Data are presented as mean ± standard deviation (SD) and error bars represent the SD among three independent replicates (*n* = 3). Normality was evaluated using the Kolmogorov–Smirnov test and one-way ANOVA tests were applied to assess differences between formulations at each time point. Statistically significant differences in PTX release were observed during the early phase (1–3 h), with HSA-PTX-TMAB NPs exhibiting a faster release rate (*p* < 0.05). At later points (≥5 h), no significant differences were detected as both systems reached a release plateau.

The in vitro release profiles (Figure 5) showed that HSA-PTX NPs released approximately 50% of the PTX within 3 h, whereas the HSA-PTX-TMAB NPs reached the same release fraction within 1 h. Both systems reached a plateau after 5 h, with no further drug release (*p* > 0.05).

Fitting of the data indicated that HSA-PTX release followed a Higuchi model (R^2^ = 0.9779), while that of HSA-PTX-TMAB followed a Korsmeyer–Peppas model (R^2^ = 0.9540; n = 1.6240) [65], comprising a super case II transport mechanism (Table 5).

### 3.4. In Vitro Cytotoxicity Assay

**Figure 6:**
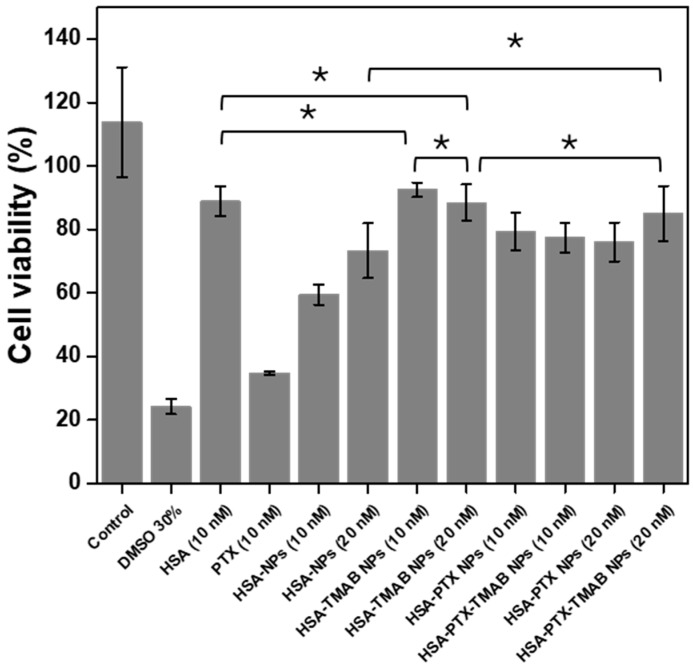
Effect of human serum albumin (HSA), human serum albumin nanoparticles without paclitaxel (HSA NPs), human serum albumin–paclitaxel nanoparticles (HSA-PTX NPs), trastuzumab-decorated human serum albumin nanoparticles without paclitaxel (HSA-TMAB NPs), and trastuzumab-decorated human serum albumin–paclitaxel nanoparticles (HSA-PTX-TMAB NPs) on A549 cells after 18 h of treatment. Data are presented as mean ± standard deviation (SD) and error bars represent the SD among three independent replicates (*n* = 3). Normality was assessed using the Kolmogorov–Smirnov test and statistical differences were evaluated using one-way ANOVA. Three distinct response groups were identified: the control exhibited significantly higher viability than all treated conditions (*p* < 0.05); HSA NPs and HSA-TMAB NPs showed the lowest viability and did not differ significantly from each other; and all PTX-containing formulations and remaining treatments formed a statistically similar group with intermediate viability values (*p* > 0.05). In the graph, bars marked with an asterisk (*) indicate groups that do not present statistically significant differences (*p* > 0.05).

Figure 6 shows the viability of A549 cells after 18 h exposure to the various nanoparticle formulations. HSA NPs reduced viability by more than 40%, while native HSA reduced it by approximately 10%. HSA-TMAB NPs did not show a significant decrease in viability. Both of the HSA-PTX and HSA-PTX-TMAB formulations produced around a 20% reduction in cell viability, with no significant differences between them (*p* > 0.05).

## 4. Discussion

### 4.1. Structural Characterization

Thermal denaturation of HSA induced clear secondary structure transitions, evidenced by increased random coil and intermolecular β-sheet content in the FTIR spectra [75]. These changes reflect the disruption of hydrogen bonding and the exposure of hydrophobic amino acid residues, which drive the self-assembly process that leads to HSA-PTX NP formation. The increase in intermolecular β-sheet structures stabilizes the matrix through hydrogen bonding, thereby enhancing mechanical robustness and drug-loading capacity [76,77].

An increase in intermolecular β-sheet structures enhances NPs’ rigidity through hydrogen-bonded crosslinking, whereas a rise in random coils reflects structural loosening required for hydrophobic accommodation [78]. These FTIR results are consistent with previous reports on polypeptide- and protein-based NPs, where heat-induced unfolding facilitates the formation of stable, drug-loaded assemblies in the absence of chemical crosslinkers [79].

### 4.2. Physicochemical Characterization

The moderate increase in mean diameter observed for the HSA-PTX-TMAB formulation compared to that of the HSA-PTX formulation indicates successful TMAB adsorption without a significant effect on the mean diameter. In contrast, the larger size and higher PDI of HSA-TMAB NPs can be attributed to electrostatic interactions occurring near the isoelectric point of the antibody [80], which promote protein clustering and increase heterogeneity. Compared with self-assembling peptide-based systems such as FER-8 [81], which form nanostructures with characteristic dimensions below 500 nm at pH 7.4, the albumin-based NPs reported here are markedly smaller (~287–307 nm), suggesting a more compact nanoscale assembly under physiological conditions.

The decrease in ζ-potential after TMAB adsorption reflects partial neutralization of negatively charged albumin domains, an effect wherein the antibody binds electrostatically to the HSA surface [45]. Despite this reduction, both formulations maintained ζ-potential values associated with stable colloidal dispersions [82,83]. Stability studies conducted over 30 days at 4 °C demonstrated the robustness of the thermally driven assembly, with no detectable changes in size or dispersity. The slight decrease in ζ-potential for the HSA-PTX-TMAB NPs indicates minor surface rearrangements over time, which may be attributed to partial TMAB desorption or reorganization at the nanoparticle interface [84].

These findings highlight the ability of the thermally induced conformational state of HSA to promote nanoparticle assembly without the use of glutaraldehyde for crosslinking stabilization [36]. The consistently low PDI values and negative ζ-potential confirm that the unfolding-driven self-assembly leads to a reproducible colloidal system.

The SEM micrographs corroborated the formation of the spherical, smooth NPs characteristic of self-assembled HSA systems, with no structural collapse after lyophilization [85]. In contrast, several peptide-based NP systems reported in the literature tend to self-assemble into fibrillar or rod-like nanostructures rather than spherical particles, reflecting fundamental differences in the underlying assembly mechanisms [81,86]. The spherical morphology is particularly relevant for biomedical applications, as it is generally associated with improved biocompatibility, reduced immune recognition, and more favorable interactions within biological environments [87].

### 4.3. Drug Loading and Release Behavior

The high drug association efficiency in both the HSA-PTX and HSA-PTX-TMAB systems confirms strong hydrophobic interactions between PTX and the thermally unfolded HSA matrix [33]. The obtained *%DAE* values are comparable to those reported for amphiphilic polypeptide-based nanocarriers. For example, a polypeptide–chitosan nanoparticle system has been reported to achieve a PTX *%DAE* of 84.6% with a high %DL of 34.7% [88], while other polypeptide formulations exhibit a *%DAE* of 34.28% and a %DL of 29.61% [89].

In contrast, the HSA–PTX formulations developed in this study exhibited markedly higher association efficiencies, reaching 96.4% for HSA–PTX and 98.2% for HSA–PTX–TMAB, albeit with lower %DL values (8.9% and 7.4%, respectively). Significantly, these loading capacities fall within the range reported for clinically approved nab–paclitaxel formulations [90,91], supporting the suitability of the thermally driven self-assembly strategy for efficient paclitaxel incorporation and translational relevance.

Regarding drug release, modeling revealed distinct kinetic behaviors between the formulations: HSA-PTX follows a diffusion-controlled Higuchi mechanism [92], while HSA-PTX-TMAB exhibits a super case II transport behavior described by the Korsmeyer–Peppas model [93,94]. This transition suggests that TMAB adsorption induces structural relaxation and rearrangement within the albumin matrix, altering diffusion pathways. Mechanistically, the faster PTX release observed in the HSA-PTX-TMAB NPs was consistent with competitive interactions at domain IIIA near Sudlow site II of HSA, where TMAB and PTX may compete for binding [95], facilitating the displacement of weakly associated PTX and contributing to the burst phase.

Consistent with this interpretation, the release profiles of both the HSA–PTX and HSA–PTX–TMAB systems exhibited rapid initial release, followed by an apparent stabilization phase. This behavior aligns well with that of several protein- and polypeptide-based PTX delivery systems reported in the literature. Comparative studies evaluating PTX-loaded submicron particles based on serum albumin, whey protein, casein, and Pluronic have shown initial burst releases of approximately 45–55% within the first 4 h, followed by plateau values of 60–70% over 48–72 h. Within this context, the ~50% PTX release observed within 1–3 h for the HSA-PTX and HSA-PTX-TMAB formulations developed here is comparable to albumin and whey-based systems [96].

To further contextualize these results, the reported aqueous solubility of PTX in PBS, pH 7.4, ranges from approximately 0.3 to 30 µg/mL [97]. Considering the total amount of PTX loaded in the dialysis device (0.347 mg dispersed in 110 mL of release medium), the maximum theoretical PTX concentration in the external medium would be approximately 3.15 µg/mL. This value lies within the reported solubility range, indicating that near-sink conditions were largely maintained under the selected experimental setup. In addition, at each sampling time point, withdrawn aliquots were replaced with an equal volume of fresh PBS to preserve constant volume and concentration gradients throughout the release experiment.

PBS-based release conditions were intentionally selected to enable direct comparison with previously reported albumin and PTX NP delivery systems, which commonly employ similar methodologies [98,99]. Within this comparative framework, the relative differences observed during the early stages of release between the non-decorated and antibody-decorated NPs remain meaningful and reflect genuine differences in nanoparticle structure and drug–carrier binding equilibria.

From a therapeutic perspective, the rapid initial release followed by subsequent stabilization observed for both formulations may be advantageous in applications where early local drug availability is desired while prolonged systemic exposure is not required. Such release behavior may be particularly relevant for treatment strategies in which rapid attainment of therapeutic concentrations at the target site enhances efficacy without increasing systemic toxicity. In support of this rationale, pharmacokinetic studies have shown that non-intravenous administration of paclitaxel can result in higher drug accumulation in lung tissue and reduced systemic distribution compared to intravenous delivery [71]. Accordingly, the release profile observed for the HSA-PTX and HSA-PTX-TMAB NPs, characterized by fast availability and matrix stabilization, may be well suited for therapies that prioritize localized exposure over sustained systemic circulation.

### 4.4. Cytotoxicity Analysis

The cytotoxicity assay was conducted with a short exposure period (18 h) to evaluate the potential acute, non-specific cytotoxic effects associated with the NP formulations, rather than their long-term antiproliferative efficacy. Previous studies have shown that, in A549 cells, low PTX concentrations primarily induce modulation of cell-cycle regulatory pathways rather than immediate cell death [100]. Based on this established cellular response, the 18 h exposure time was selected to capture early cellular effects while avoiding overestimation of short-term cytotoxicity. In this context, the modest reductions in cell viability observed for the PTX formulations at 18 h are expected and should not be interpreted as a lack of pharmacological potential.

The cytotoxicity trends observed in A549 cells are consistent with previously reported studies on HSA-PTX NPs evaluated at comparable drug concentrations and short exposure times [88]. In such systems, PTX concentrations in the range of 8 µg/mL have been shown to induce only moderate reductions in cell viability (~70–75%) after 24 h, while more pronounced cytotoxic effects require either higher PTX doses or longer incubation periods. In the present study, the use of PTX concentrations of 4 and 8 µg/mL, combined with a shorter exposure time (18 h) and the distinct proliferation kinetics of A549 cells, reasonably accounts for the observed viability levels.

The moderate cytotoxic effects observed across the formulations are consistent with NP systems that exhibit strong colloidal stability and controlled release rather than rapid intracellular delivery. This behavior aligns with the formulation-driven nature of the present work, in which the structural features governing PTX loading and release do not appear to compromise the NPs’ integrity or induce unintended toxicity.

For PTX-loaded systems, the relatively modest reduction in cell viability, despite high drug association efficiency and faster release in the TMAB-decorated formulation, may be influenced by the formation of protein corona upon exposure to the biological media [101,102]. These biomolecule–NP complexes can delay effective intracellular delivery of PTX [103,104], thereby attenuating early cytotoxic responses. Importantly, this behavior is compatible with applications requiring moderated release profiles, as it may prolong local drug retention and minimize systemic exposure [103].

In the case of blank has NPs, the reduction in cell viability may relate to general nanoparticle internalization pathways mediated by albumin-interacting receptors such as FcRn [105,106], rather than to specific biological activity of the carrier. Thermal processing of HSA has been reported to increase β-sheet content and protein rigidity, which under certain conditions may influence cell membrane interactions [107,108,109]; however, these effects appear limited within the context of the current formulation. Conversely, the negligible activity observed for HSA-TMAB could result from lower cellular uptake of antibody-decorated aggregates stabilized by hydrophobic, hydrogen-bond, and ionic interactions [45,110].

Overall, the cytotoxicity outcomes support the conclusion that the physicochemical properties optimized in this study, particularly the structural stability of the albumin matrix and the PTX release mechanisms, do not translate into unintended cytotoxic responses under the tested conditions. While a comprehensive evaluation of efficacy will require extended exposure times and receptor-specific uptake studies, the present results are sufficient to validate the performance of the formulation from a materials and drug-delivery standpoint.

## 5. Conclusions

This study demonstrates the successful design of HSA-based nanoparticles for paclitaxel (PTX) delivery using a thermally driven self-assembly approach, followed by non-covalent trastuzumab (TMAB) adsorption for surface decoration. Both the HSA–PTX and HSA–PTX–TMAB formulations exhibited high drug association efficiency (*%DAE*) and loading capacity (*%LC*) values within the range reported for clinically relevant albumin-based PTX systems, together with robust colloidal stability, supporting the reproducibility of the unfolding-mediated assembly process. TMAB decoration accelerated the early-stage release of PTX. It was associated with a change in the fitted release mechanism, which may reflect TMAB-induced matrix relaxation and competitive interactions at HSA drug-binding regions.

Cytotoxicity in A549 cells after a short exposure (18 h) showed modest, non-differential effects across PTX-loaded formulations. This outcome is consistent with the short-term nature of the assay, its controlled release behavior, and the potential influence of protein corona formation on early cellular uptake. Therefore, it should not be interpreted as a lack of pharmacological potential. Collectively, these characteristics may be advantageous for applications that benefit from rapid initial drug availability followed by stabilization, particularly in localized delivery strategies where prolonged systemic exposure is not required.

Overall, the simplicity, mild processing conditions, and scalability of this thermally driven method highlight its potential as a foundation for targeted PTX delivery, including pulmonary or other localized approaches. Future work should include in vivo pharmacokinetic and biodistribution studies, quantitative confirmation of TMAB surface binding and HER2-mediated uptake, and extended antiproliferative assays (72–96 h) using cell models with high and low/negative HER2 expression to clarify selectivity and long-term efficacy. In addition, exploration of alternative functionalization techniques, such as covalent antibody conjugation, may also improve tumor selectivity and therapeutic efficacy.

## Figures and Tables

**Table 1:** The secondary structure of HSA and the second derivative of the amide I band.

Wavenumber, ν (cm^−1^)	Range (cm^−1^)	Secondary Structure
1618	1610–1619	Intermolecular β-sheet
1628, 1638, 1691	1620–1639, 1689–1695	β-sheet
1640, 1649	1640–1649	Random coil
1650, 1657	1650–1660	α-helix
1673, 1681	1660–1689	β-turn

The relationship between the secondary structure of HSA and the second derivative of amide I [72].

**Table 2:** The fraction contents of diverse secondary structures of native HSA (control) and denatured HSA (%).

	α-Helix %	β-Sheet %	Random Coil %	β-Turn %	Intermolecular β-Sheet %
Native HSA	27.3	61.1	0	10.9	0.8
HSA-PBS 70 °C *	24.1	41.9	28.7	2.7	2.6

The fractional composition of the main secondary structure elements of native HSA and thermally denatured has, determined from deconvolution of the amide I band (1600–1700 cm^−1^). The decrease in α-helix content and the concomitant increase in random coil and intermolecular β-sheet structures confirm the conformational rearrangement of HSA after thermal treatment at 70 °C in PBS. * HSA-PBS 70 °C corresponds to the denatured HSA in phosphate-buffer saline.

**Table 3:** Mean diameter, polydispersity index (PDI), and ζ-potential.

Formulation	Mean Diameter (nm)	PDI	ζ-Potential (mV)
HSA-PTX NPs	287.1 ± 12.9	0.10 ± 0.01	−36.5 ± 1.4 *
HSA-TMAB	379.1 ± 3.3 *	0.30 ± 0.23	−21.1 ± 5.0
HSA-PTX-TMAB NPs	306.9 ± 42.2	0.23 ± 0.13	−24.0 ± 1.4

The data are presented as the mean ± standard deviation (SD) from three independent experiments (*n* = 3). Normality was assessed using the Kolmogorov–Smirnov test. For normally distributed datasets, one-way ANOVA was applied. Statistical analysis showed that the HSA-TMAB formulation exhibited significant differences in mean diameter compared with the HSA-PTX NPs (*p* < 0.05). No significant differences were observed among formulations in the PDI values. For ζ-potential, the HSA-PTX NPs displayed significantly more negative values compared with the other systems (*p* < 0.05). Asterisks (*) denote statistically significant differences (*p* < 0.05).

**Table 4:** Drug association efficiency (*%DAE*), loading capacity (*%LC*), paclitaxel loading (µg PTX/mg HSA), and orientative trastuzumab loading (µg TMAB/mg HSA) for HSA-PTX and HSA-PTX-TMAB nanoparticles.

Formulation	*%DAE*	*%LC*	PTX Loading (µg PTX/mg HSA)	TMAB Loading (µg TMAB/mg HSA) *
HSA-PTX NPs	96.4 ± 2.1	8.9 ± 2.9	128.5 ± 2.8	—
HSA-PTX-TMAB NPs	98.2 ± 3.5	7.4 ± 1.5	130.9 ± 4.6	250 *

The data are presented as the mean ± standard deviation (SD) from three independent experiments (*n* = 3). The normality was evaluated using the Kolmogorov–Smirnov test, followed by one-way ANOVA for normally distributed datasets. The drug association efficiency (*%DAE*), loading capacity (*%LC*), and PTX loading (µg PTX/mg HSA) did not show statistically significant differences between human serum albumin–paclitaxel nanoparticles (HSA-PTX NPs) and human serum albumin–paclitaxel–trastuzumab nanoparticles (HSA-PTX-TMAB NPs) (*p* > 0.05). The TMAB loading value (µg TMAB/mg HSA) is provided as an orientated estimate based solely on the amount of antibody added during formulation assuming 100% adsorption efficiency; therefore, it does not represent an experimental quantification of antibody bound to the nanoparticle surface *.

**Table 5:** Kinetic release parameters at pH 7.4 for PTX from HSA-PTX and HSA-PTX-TMAB NPs obtained after fitting data to various mathematical models.

Model	HSA-PTX			HSA-PTX-TMAB		
	K	R^2^	n	K	R^2^	n
Zero order	11.682	0.9072	-	14.648	0.8226	-
First order	0.155	0.7324	-	0.1287	0.7367	-
Higuchi	26.135	0.9779	-	33.317	0.9164	-
Hixon-Crowell	−0.2542	0.9451	-	−0.4048	0.8814	-
Korsmeyer-Peppas	−0.2539	0.9141	1.7923	−0.5029	0.9540	1.6240

R^2^, coefficient of determination; n, diffusion coefficient of the Korsmeyer–Peppas model [65].

## Data Availability

The raw data supporting the conclusions of this article will be made available by the authors on request.
